# Whole-Genome Resequencing of a Cucumber Chromosome Segment Substitution Line and Its Recurrent Parent to Identify Candidate Genes Governing Powdery Mildew Resistance

**DOI:** 10.1371/journal.pone.0164469

**Published:** 2016-10-20

**Authors:** Qiang Xu, Yang Shi, Ting Yu, Xuewen Xu, Yali Yan, Xiaohua Qi, Xuehao Chen

**Affiliations:** School of horticulture and plant protection, Yangzhou University, Jiangsu, 225009, China; US Department of Agriculture, UNITED STATES

## Abstract

Cucumber is an economically important vegetable crop worldwide. Powdery mildew (PM) is one of the most severe diseases that can affect cucumber crops. There have been several research efforts to isolate PM resistance genes for breeding PM-resistant cucumber. In the present study, we used a chromosome segment substitution line, SSL508-28, which carried PM resistance genes from the donor parent, JIN5-508, through twelve generations of backcrossing with a PM-susceptible inbred line, D8. We performed whole-genome resequencing of SSL508-28 and D8 to identify single nucleotide polymorphisms (SNPs), and insertions and deletions (indels). When compared against the reference genome of the inbred cucumber line 9930, a total of 468,616 SNPs and 67,259 indels were identified in SSL508-28, and 537,352 SNPs and 91,698 indels were identified in D8. Of these, 3,014 non-synonymous SNPs and 226 frameshift indels in SSL508-28, and 3,104 non-synonymous SNPs and 251 frameshift indels in D8, were identified. Bioinformatics analysis of these variations revealed a total of 15,682 SNPs and 6,262 indels between SSL508-28 and D8, among which 120 non-synonymous SNPs and 30 frameshift indels in 94 genes were detected between SSL508-28 and D8. Finally, out of these 94 genes, five resistance genes with nucleotide-binding sites and leucine-rich repeat domains were selected for qRT-PCR analysis. This revealed an upregulation of two transcripts, *Csa2M435460*.*1* and *Csa5M579560*.*1*, in SSL508-28. Furthermore, the results of qRT-PCR analysis of these two genes in ten PM resistant and ten PM susceptible cucumber lines showed that when exposed to PM, Csa2M435460.1 and Csa5M579560.1 exhibited a higher expression level of resistant lines than susceptible lines. This indicates that Csa2M435460.1 and Csa5M579560.1 are candidate genes for PM resistance in cucumber. In addition, the non-synonymous SNPs in *Csa2M435460*.*1* and *Csa5M579560*.*1*, identified in SSL508-28 and D8, might be the key to high PM-resistance in SSL508-28.

## Introduction

Cucumber (*Cucumis sativus* L., 2n = 2x = 14) is one of the most important vegetable crops worldwide. In China, production of the cucumber accounted for more than 1 million hectares of land, with 54.32 million tons of cucumber produced in 2013. (http://www.faostat3.fao.org). Powdery mildew (PM) is one of the most serious diseases that can affect cucumber crops, causing severe losses in yield and quality. The traditional way to control PM is by applying protective fungicides [1]; however, their extensive use is not only increase selection pressure on pathogen populations to adapt and acquire increasing levels of fungicide resistance but it also detrimental to the environment and human health[2]. The most effective way to control the disease is by breeding PM-resistant cultivars.

Many studies have focused on detecting PM-resistant quantitative trait loci (QTLs) in cucumber. Sakata et al. [3] first used a population of F7 recombinant inbred lines to map QTLs for PM resistance in cucumber, and detected six QTLs. Liu et al. [4] identified five QTLs in two environments, using 130 F2:3 lines. de Ruiter et al. [5] identified two PM resistance QTLs in an F2 population. Zhang et al. [6] detected four linked PM resistance QTLs using F2 and F2:3 populations. Fukino et al. [7] identified nine QTLs for PM resistance in a population of 111 recombinant inbred lines. More recently, Nie et al. [1] used secondary segregating populations to map a 170-kb region PM resistance QTL named *pm5*.*1*. Although such findings have provided insight into the genetic control of PM resistance in cucumber, the detailed mechanisms underlying PM resistance remain unclear. Importantly, the molecular markers identified in these studies are not breeder friendly [8].

All plants have evolved various defense mechanisms against a diverse number of pathogens that include bacteria, fungi, viruses, and nematodes. The most effective mechanisms protect against specific pathogen species; such specific resistance is conveyed by resistance genes (R-genes) [9]. In this mechanism, R-gene-activated resistance is known as effector-triggered immunity and the recognized gene products of the pathogens are called avirulence proteins [10]. R-genes are the specificity determinants of plant effector-triggered immunity [11] and the most numerous R-gene class is represented by members of a gene family containing a nucleotide-binding site (NBS) and a leucine-rich repeat (LRR) domain [12]. R-genes encoding NBS and LRR domains are thought to be involved in pathogen recognition and subsequent initiation of defense responses [13]. Studies of such genes in plants such as *Arabidopsis thaliana*, rice, and wheat [14–16] have significantly increased our understanding of the molecular genetic basis of host resistance in the context of PM infection. Coleman et al. [17] located a PM R gene named Ren1 with an NBS-LRR domain in grape. More recently, Jordan et al. [18] reported that the gene TmMla1, with an LRR domain, was involved in resistance to a species of PM in barley. However, to date, there have been few studies of NBS-LRR genes in cucumber during PM infection. The draft genome of the Northern China type inbred cucumber line 9930 was released as a reference genome sequence using next-generation sequencing technology [19]. This technology allows rapid resequencing of the whole genome in multiple individuals and comparison to a reference genome, to detect genetic variants between the different samples. Such variations reveal potential functional information, often affecting gene structure and contributing to interspecies phenotypic traits and adaptations [20]. To date, resequencing technology has been used to study individual variations in cucumber, maize, rice, and other plants, and has made a significant contribution to germplasm evolution and molecular breeding [21–25]. Therefore, re-sequencing technology is an effective tool by which to identify genetic variations which can cause phenotypic differences between cucumbers.

Chromosome segment substitution lines (CSSLs) have played an important role in uncovering QTLs in many crops [4, 26]. CSSLs are produced by crossing and backcrossing, and using molecular marker-assisted selection to build a series of fragments covering the entire genome of the crop. CSSLs have a high level of genetic background uniformity with the recurrent parent, so any phenotypic variations between SSLs and recurrent parent could be associated with the substituted segments. In our previous study, the PM resistant line SSL508-28 introgressed segments from JIN5-508 (highly PM-resistant, sprawl), and a line with a different genetic background, D8 (PM-susceptible, dwarf), was developed using marker-assisted selection with sequence characterized amplified regions, and simple sequence repeats [27].

In the present study, we performed whole-genome resequencing in the SSL508-28 and its recurrent parent, D8, to uncover non-synonymous SNPs and frameshift indels. Five of these substituted genes with NBS-LRR or LRR domains were then selected for qRT-PCR after inoculation with the PM pathogen in D8 and SSL508-58. Two genes, Csa2M435460.1 and Csa5M579560.1, were identified as potential candidates for PM resistance in cucumber.

## Materials and Methods

### Plant Materials

The SSL508-28 line was derived by marker-assisted backcrossing [27] with an inbred line JIN5-508 (PM-resistant, sprawl) as the donor, and D8 (PM-susceptible, dwarf) as the recipient parent. Following an initial cross using D8 as the female, backcrossing to D8 was performed for 12 generations. At each generation, a sequence-characterized amplified region marker was used to select dwarf plants for backcrossing with D8, and then a sequence-characterized amplified region marker linked with PM resistance (obtained in our earlier study in JIN5-508) was used to select disease-resistant plants after inoculating these dwarf plants with the PM pathogen [27]. Finally, 17 plants were developed from the crosses and generated 17 families, with a total of 449 plants, by self-fertilization, for homozygous CSSL selection. Among these CSSLs, two resistant dwarf lines, SSSL0.7 and SSL508-28, which carried a chromosome fragment from JIN5-508, showed high resistance to PM [27]. Resistant genes in SSSL0.7 have been reported previously [28].

### Confirmation of PM Infection

PM-resistance experiments in JIN5-508, D8 and SSL508-28 were conducted across three growing seasons: autumn 2014, spring 2015, autumn 2015. The PM pathogen was isolated from the leaves of diseased cucumber plants grown in a greenhouse at Yangzhou University. Cucumber seedlings at the two-true-leaf stage were inoculated with the pathogen by spraying them with a spore suspension (10^6^ spores/ml). After inoculation, the seedlings were kept in a greenhouse under controlled conditions (30°C day/25°C night; 80 ± 15% relative humidity). Fifteen days later, the disease severity of three leaves from each plant was scored visually on a scale of 0–5, where 0 = no symptoms; 1 = infection area <30%; 2 = 30–59%; 3 = 60–79%; 4 = ≥80%; and 5 = death of leaf. Disease index was calculated according to the following formula: ∑ (disease scale × number of leaves of that scale)/(number of leaves inoculated × highest disease grade) × 100.

### Sample Preparation and Resequencing

The resequencing of SSL508-28 and D8 was performed by Biomarker Technologies (Beijing, China). Total genomic DNA of SSL508-28 and D8 was extracted from the young leaves and then fragmented randomly. DNA fragments of interest were extracted using agarose gel electrophoresis, adapters were ligated to both ends of the target DNA fragments, and DNA clusters were prepared for paired-end sequencing using the Illumina HiSeq 2500 ultra-high-throughput sequencing system.

### Read Mapping and Analysis of SNPs/Indels

The raw data were corrected and filtered [29–31] to obtain high quality, clean data from D8 and SSL508-28. Using Burrows–Wheeler Aligner software [32], these data were mapped onto the 9930 cucumber reference genome. The sequence alignment map format was converted to binary alignment map files (SAMtools version 0.1.18; http://sourceforge.net). Picard tools were used to remove duplicate reads, and GATK tools (version 1.5; http://www.broadinstitute.org) were used to conduct insertion and deletion (indel) realignment and base quality recalibration of the binary alignment map files. A set of high-quality SNPs were obtained by variant quality score recalibration, using GATK to filter high-quality indels with the command QD < 2.0, ReadPosRankSum < −20.0 FS > 200.0. In the final step, SNPs and indels were identified after removal of variants with depth coverage <10 and those located outside exome-capture regions. The distribution of SNPs and indels on each cucumber chromosome was visualized using Circos [33].

### Variations and Annotation of SNPs and Indels in D8 and SSL508-28

Variations of SNPs and indels obtained from the read mapping results of D8 and SSL508-28 were further compared to identify differences in SNP and indel variation between the two lines. The resulting variants file was processed using SnpEff software [34] to identify the gene positions, identifiers, and functional descriptions. Synonymous and non-synonymous substitutions were identified as described previously [35].

### Candidate Gene Detection

The SNPs and indels in a coding sequence cause the non-synonymous and frameshift mutations that alter the amino acid sequence of a protein and may lead to a change in gene function. Therefore, the 9930 reference genome was used to identify differences in non-synonymous SNPs and frameshift indels between D8 and SSL508-28. The putative functions of the genes containing these differential variations were predicted using Blastx at the NCBI (National Center for Biotechnology Information) website (http://blast.ncbi.nlm.nih.gov). The genes containing NBS, LRR, or NBS-LRR domains were considered candidate genes for PM resistance and selected for further validation.

### Quantitative Reverse-Transcription (qRT)-PCR Analysis of Candidate Genes

qRT-PCR was used to validate the genes related to PM resistance. Total RNA was isolated from D8 and SSL508-28 leaf samples frozen at 0, 12, 24, 36, 48 and 72 h after inoculation with mildew pathogen, using RNAiso Plus (Takara, Dalian, China). Total RNA was used for first-strand cDNA synthesis according to a PrimeScript RT reagent kit (Takara). Primers were designed from a 3′untranslated region for candidate genes using Primer Premier 5.0. qRT-PCR was performed using a SYBR PrimeScript RT-PCR kit (Takara), according to the manufacturer’s instructions, and detected using an iQ5 real-time PCR detection system (Bio-Rad, Hercules, CA, USA) in 25 μL reactions. The PCR conditions for all genes were: 30 s at 95°C, 40 cycles of 5 s at 95°C and 30 s at 55°C, and 30 s at 72°C. Relative expression levels of the genes were determined using the 2^−ΔΔCt^ method after three replications. To identify the candidate genes for resistance, 80 different cucumber lines from our lab were grown in a greenhouse at Yangzhou University in May 2016, and PM conidial water suspension (10^6^ spores/ml) was sprayed evenly on the cotyledons and true leaves when the seedlings were at the two-true-leaf stage. The PM disease index of these 80 cucumber lines was calculated 15 days after inoculation. Then ten PM resistant lines(Jin5-508,Baiguifei,JY35,Riyin,Biyu,Haiyang,Zaoer-N,BY2,GY2,Xiafeng;DI<2) and ten PM susceptible lines(EP6392,Superlna,Axin,Yanbai,Xiaoye,EP6411,Rijiecheng,Yihuang,Zaokang,S-No.70;DI>15) were selected to performqRT-PCR analysis of the candidate genes.

### LRR Domain Detection, and Validation of Non-Synonymous SNPs in PMR-Related Genes, in D8 and SSL508-28

Protein sequences of the candidate genes were extracted from the 9930 (Version 2.0) reference sequences, and characterized using Pfam version 26.0. SMART (http://smart.embl-heidelberg.de/) was used to confirm the LRR domains. To verify the presence of the non-synonymous SNPs in PM resistance-related genes between D8 and SSL508-28, the genes were cloned in each line. Primers were designed in Primer 5.0. PM resistance-related gene sequences were amplified by PCR, using genomic DNA from D8 and SSL508-28. The PCR amplification reactions were carried out using Ex Taq DNA polymerase (Takara) in 25 μl samples, according to the manufacturer’s instructions. PCR was performed in a thermal cycler (Bio-Rad) under the following conditions: 30 s at 95°C, 40 cycles of 5 s at 95°C, 30 s at 55°C, and 30 s at 72°C, and a final extension of 10 min at 72°C. The PCR products were resolved on 1.2% agarose gel and the amplified PCR fragments were eluted and purified for sequencing.

## Results

### PM Resistance in SSL508-28

**Fig 1:**
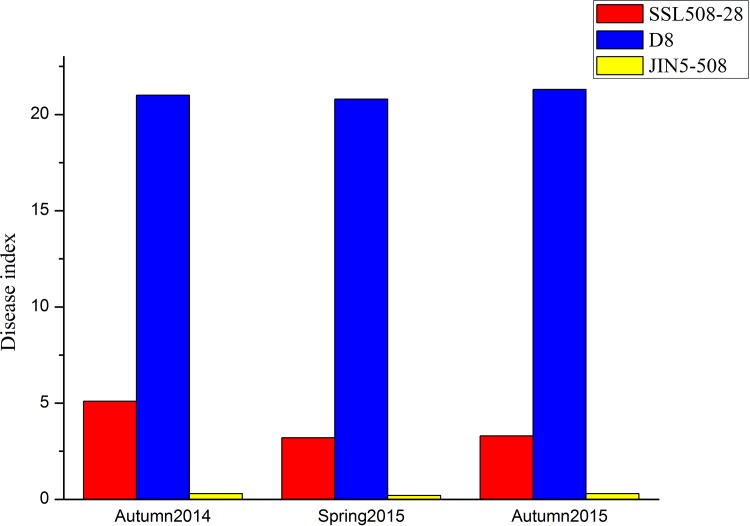
The Disease index of SSL508-28, D8 and JIN508-28 in three seasons (autumn2014, spring 2015, autumn 2015).

**Fig 2:**
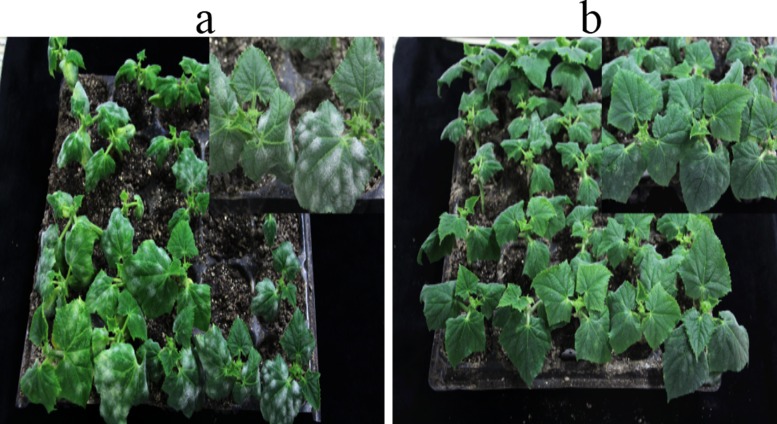
Performance of powdery mildew resistance in the seedlings of D8(a) and SSL508-28(b).

The resistance levels of SSL508-28 and its parental lines, JIN5-508 and D8, were investigated in 5-day-old seedlings in autumn 2014, spring 2015 and autumn 2015. The disease index of JIN5-508 in each season was, respectively, 0.3, 0.2, and 0.3; in D8, the disease indexes were 21.0, 20.8, and 21.3; and in SSL508-28 they were 5.1, 3.2, and 3.3 (Fig 1.). The level of resistance in SSL508-28 was slightly lower than its original donor Jin5-508, but significantly higher than that of D8. These results suggest that SSL508-28 introgressed PM resistance genes from JIN5-508 in the D8 genetic background (Fig 2.).

### Whole-Genome Resequencing

Whole-genome sequencing of SSL508-28 and D8 generated 24 gigabases of raw sequence data. Then 60,108,402 and 65,775,632 short reads (raw sequence data) were obtained for SSL508-28 and D8, respectively and, after correcting and filtering the data, we obtained 55,665,329 and 61,748,517 clean reads from SSL508-28 and D8, respectively. In SSL508-28, 70.01% of the clean reads were aligned with the 9930 reference sequence and the resequencing depth was 24×; in D8, 73.09% clean reads were mapped to the 9930 reference sequence and the effective depth was 35×. Only 71.5% clean reads were aligned with the 9930 reference sequence; this may be due to the sample genomes being very different from the 9930 reference sequence. Sequencing coverage of 9930 in SSL508-28 and D8 was 82.76% and 92.41%, respectively (Table 1).

**Table 1:** Summary statistics of D8 and SSL508-28 resequencing.

Sample	Raw Reads	Clean Reads	Mapped (%)	Ave depth	Coverage (%)
SSL508-28	60,108,402	55,665,329	70.01	24	82.76
D8	65,775,632	61,748,517	73.09	35	92.41

### Identification and Characterization of SNPs and Indels

A total of 468,616 and 537,352 SNPs were identified in SSL508-28 and D8, respectively, by comparison with the reference 9930 genome. In SSL508-28, there were 10,967 heterozygous SNPs and 457,649 homozygous SNPs. The number of transition (Ti) and transversion (Tv) SNPs for SSL508-28 were 285,700 and 182,916, respectively, and 322,520 and 214,832 for D8. The Ti/Tv ratio was 1.56 for SSL508-28 and 1.5 for D8 (Tables 2 and 3). From the total number of indels identified, 50,087 insertions and 49,260 deletions were detected in SSL508-28, and 67,323 insertions and 65,222 deletions were detected in D8. There were 96,424 heterozygous indels and 2,923 homozygous indels in SSL508-28, and 128,453 heterozygous indels and 4,092 homozygous indels in D8.

**Table 2:** Numbers and categories of SNPs identified in SSL509-28 and D8 by comparison against the 9930 genome.

Sample	SNP number^a^	Transition^b^	Trans- version^c^	Heterozygosity^d^	Homozygosity^e^	Ti/Tv^f^
SSL508-28	468,616	285,700	182,916	10,967	457,649	1.56
D8	537,352	322,520	214,832	12,065	525,287	1.5

^a^Total number of SNPs.

^b^Number conversion into same type of base.

^c^Number conversion into different types of bases.

^d^Number of heterozygous SNPs (two or more bases).

^e^Number of homozygous SNPs (only one base).

^f^Ti, transitions (C/T and G/A); Tv, transversions (C/G, T/A, A/C, and G/T).

**Table 3:** Numbers and categories of indels detected in SSL509-28 and D8 by comparison against the 9930 genome.

Sample	Insertion number	Deletion number	Total indels	Heterozygosity	Homozygosity
SSL508-28	50,087	49,260	99,347	96,424	2,923
D8	67,323	65,222	132,545	128,453	4,092

### Genomic Distribution of SNPs and Indels across Cucumber Chromosomes

**Fig 3:**
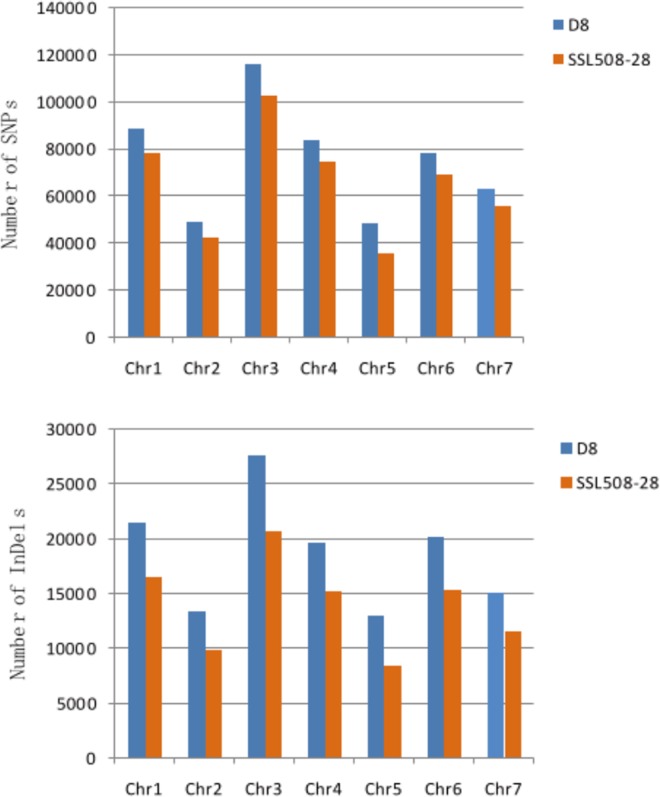
Number and distribution of SNPs and Indels detected on the cucumber chromosomes in both the cultivars (D8 and SSL508-28).

The distribution of DNA polymorphisms in SSL508-28 and D8 was compared with the cucumber 9930 reference genome. The frequency of SNPs and indels identified in both cultivars varied across seven chromosomes (Fig 3). The maximum number of SNPs and indels was detected in chromosome 3 and the minimum number in chromosome 5, for both SSL508-28 and D8 (Fig 3). The overall frequency of SNPs and indels was higher in D8 than in SSL508-28.

**Fig 4:**
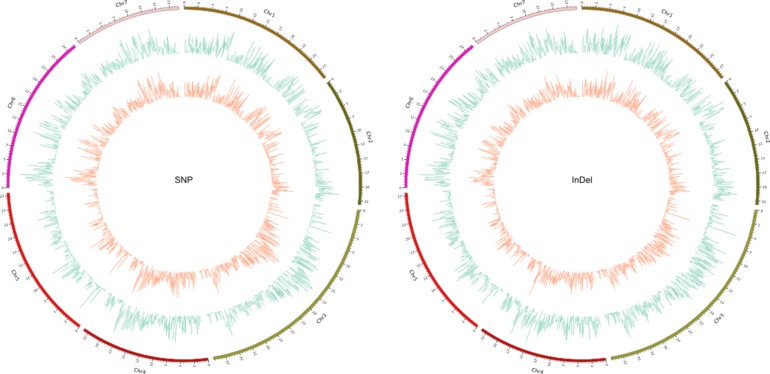
Density distribution of SNP and Indel in all the 7 chromosomes of cucumber. D8 (outer circle) and SSL508-28(inner circle).

An uneven distribution of SNPs and indels was observed between the short and long chromosome arms of the cucumbers. An average of 2417.57 SNPs per Mb was detected in SSL508-28 compared with 2855.83 in D8, and 515.26 indels per Mb in SSL508-28 compared with 686.03. SNP density was higher in chromosome 4 of SSL508-28 and D8 (3201.17 and 3580.92 SNPs per Mb, respectively) and lower in chromosome 5 (1292.24 and 1729.05 SNPs per Mb, respectively) (Fig 4). Similarly, indel density was higher in chromosome 4 of SSL508-28 and D8 (650.05 and 840.52 SNPs per Mb), and lower in chromosome 5 (302.99 and 467.47 indels per Mb). Furthermore, there was a uniform distribution of SNPs and indels in the seven chromosomes of both cultivars (Fig 4).

### Annotation of SNPs and Indels

**Fig 5:**
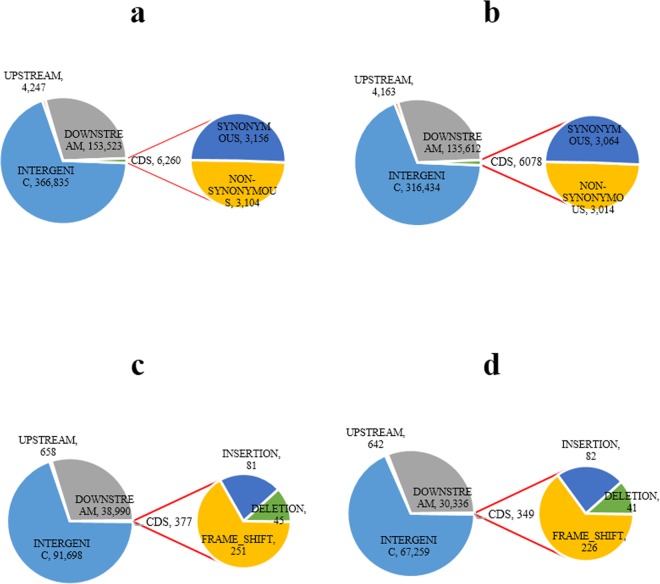
Annotation of single-nucleotide polymorphisms (SNPs) and InDels. (a,b) Distribution of SNPs in intergenic, upstream, downstream region and CDS regions for D8 and SSL508-28. (c, d) Distribution of InDels in intergenic, upstream, downstream and CDS regions for D8 and SSL508-28. The number of synonymous and non-synonymous SNPs and frameshift InDels detected within the CDS region has also been shown.

The annotated 9930 cucumber reference genome was used to reveal the distribution of SNPs and indels within the intergenic and intragenic genomic regions. Approximately 70% of SNPs and indels were identified in intergenic regions in both cultivars (Fig 5). Overall, a similar distribution pattern of SNPs and indels was observed in the entire chromosome for both cultivars. The upstream (promoter) and downstream regulatory regions had large numbers of SNPs and indels in both cultivars (30%). Moreover, only approximately 1.2% SNPs were identified in the coding sequence region in both cultivars (Fig 5). There were 3,064 synonymous and 3,014 non-synonymous SNPs in SSL508-28, and 3,156 synonymous and 3,104 non-synonymous SNPs in D8. Only 0.3% of total indels were present in the CDS region in both cultivars, and there were 226 and 251 frameshift mutation indels in SSL508-28 and D8, respectively (Fig 5).

### Genetic Variation Differences between SSL508-28 and D8

**Fig 6:**
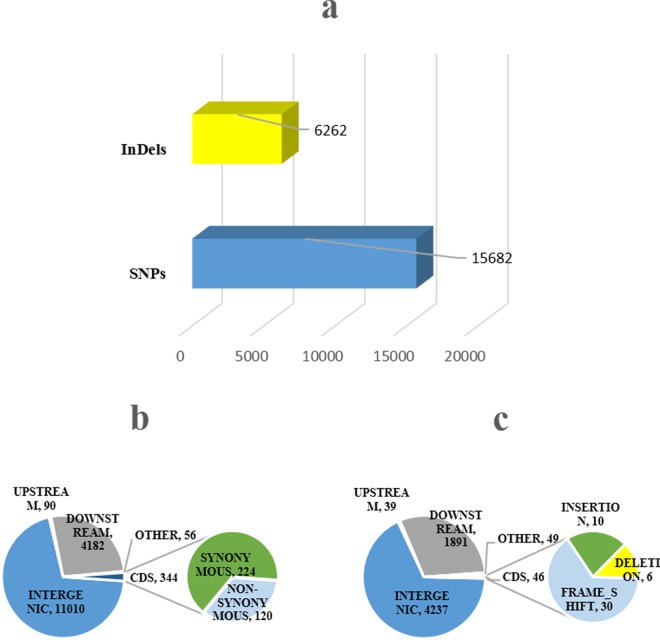
Genetic variation between SSL508-28 and D8. (a)The numbers of SNPs and InDels detected between D8 and SSL508-28. (b, c) Distribution of SNPs and InDels in intergenic, upstream, downstream region and CDS regions between D8 and SSL508-28. The number of non-synonymous SNPs and frameshift InDels detected within the CDS region has also been shown.

The different DNA variations in SSL508-28 and D8 may reflect differences in the PM-resistance genes of genome. We identified 15,682 SNP differences and 6,262 indel differences between the two lines (Fig 6), then analyzed these variations. Approximately 70% of SNPs and indels were identified in intergenic regions, whereas more than 28% of SNPs were identified in upstream (promoter) and downstream regulatory regions. Only approximately 2% of SNPs were located in coding sequence regions, and 0.36% SNPs were detected in other regions. The upstream (promoter) and downstream regulatory regions contained approximately 28% indels (Fig 6). Within the genic region, nearly 1.7% of indels were located in coding sequences. As with SNPs, the presence of indels was also observed in other regions (1%). Furthermore, 120 non-synonymous SNPs and 30 frameshift indels were identified between SSL508-28 and D8 (Fig 6). The results suggested that the difference in the genetic background between the two lines was the reason for their different levels of resistance to PM.

### Candidate Gene Identification

From the 120 non-synonymous SNPs and 30 frameshift indel variations, a total of 94 differential genes were detected between SSL508-28 and D8 (Table 4). Functional annotation of all 94 genes was performed in Blastx and then the domain organizations of each gene were investigated. Finally, one gene with leucine-rich repeat (LRR) domain and four genes with nucleotide-binding site (NBS) and leucine-rich repeat (LRR) domains were selected further analysis for identification of the candidate PM-resistance genes (Table 5).

**Table 4:** Predicted functions of genes containing non-synonymous SNPs and frameshift indels.

#	Type^a^	Gene^b^	Predicted function^c^
1	SNP	Csa1M363460.1	No hits found
2	SNP	Csa1M368490.1	No hits found
3	SNP	Csa1M574980.1	MATE efflux protein-related
4	SNP	Csa2M014330.1	No hits found
5	SNP	Csa2M026940.1	LOX1; lipoxygenase
6	SNP	Csa2M037270.1	Photosystem II CP43 chlorophyll apoprotein
7	SNP	Csa2M078060.1	No hits found
8	SNP	Csa2M264570.1	Chaperone protein dnaj 11
9	SNP	Csa2M393180.1	60S ribosomal protein L38
10	SNP	Csa2M435460.1	Disease resistance protein
11	SNP	Csa3M175740.1	Invertase/pectin methylesterase inhibitor family protein
12	SNP	Csa3M496870.1	PDR9 (pleiotropic drug resistance 9)
13	SNP	Csa3M593690.1	No hits found
14	SNP	Csa3M798610.1	Predicted protein
15	SNP/Indel	Csa3M828430.1	AAA-type ATPase family protein
16	SNP	Csa3M850670.1	No hits found
17	SNP	Csa3M856020.1	ATP synthase CF1 alpha subunit
18	SNP	Csa3M902230.1	Chitin binding peritrophin-A domain
19	SNP	Csa4M014080.1	Cysteine protease
20	SNP	Csa5M114640.1	Leucine-rich repeat transmembrane protein kinase
21	SNP	Csa5M164650.1	No hits found
22	SNP	Csa5M449150.1	No hits found
23	SNP	Csa5M454720.1	NAD-dependent malic enzyme
24	SNP	Csa5M487190.1	No hits found
25	SNP	Csa5M488760.1	Pectinesterase family protein
26	SNP	Csa5M488790.1	No hits found
27	SNP/Indel	Csa5M495930.1	Disease resistance protein
28	SNP	Csa5M495950.1	Elongation factor 1-alpha, putative
29	SNP	Csa5M497060.1	Leukotriene A4 hydrolase
30	SNP	Csa5M503560.1	Leukotriene A4 hydrolase
31	SNP	Csa5M503570.1	Predicted protein
32	SNP	Csa5M505220.1	Overexpression leads to PEL (Pseudo-Etiolation in Light) phenotype
33	SNP	Csa5M511820.1	Auxin-induced protein 5NG4, putative
34	SNP/Indel	Csa5M511830.1	No hits found
35	SNP	Csa5M511840.1	Auxin-induced protein 5NG4, putative
36	SNP	Csa5M517770.1	PQ-loop repeat family protein / transmembrane family protein
37	SNP	Csa5M523080.1	LOB domain-containing protein
38	SNP	Csa5M523090.1	Encodes oleosin 4
39	SNP	Csa5M523110.1	No hits found
40	SNP/Indel	Csa5M524810.1	Chloroplast ribosomal protein L2
41	SNP	Csa5M524820.1	Unknown protein
42	SNP	Csa5M527890.1	ATP-dependent Clp protease ATP-binding subunit Clpx, putative
43	SNP	Csa5M576690.1	No hits found
44	SNP/Indel	Csa5M576710.1	No hits found
45	SNP	Csa5M576730.1	Unknown protein
46	SNP	Csa5M577380.1	Unknown protein
47	SNP/Indel	Csa5M577450.1	Transcription factor
48	SNP/Indel	Csa5M579010.1	TIR-NBS-LRR disease resistance protein
49	SNP	Csa5M579560.1	Disease resistance family protein / LRR family protein
50	SNP	Csa5M585450.1	Protein kinase, putative
51	SNP	Csa5M587100.1	No hits found
52	SNP	Csa5M587170.1	Cleavage and polyadenylation specificity factor 73-I
53	SNP	Csa5M589970.1	Unknown protein
54	SNP	Csa5M590200.1	F-box family protein
55	SNP	Csa5M591760.1	TTN8 (TITAN8)
56	SNP	Csa5M591760.2	COP1-Interactive protein
57	SNP	Csa5M597480.1	Unknown protein
58	SNP	Csa5M600900.1	F-box/kelch protein
59	SNP	Csa5M600950.1	Pyrrolidone-carboxylate peptidase family protein
60	SNP	Csa5M601480.1	Naphthoate synthase
61	SNP	Csa5M601500.1	Galactinol synthase
62	SNP	Csa5M601530.1	bHLH transcription factor
63	SNP	Csa5M601570.1	Alcohol dehydrogenase
64	SNP	Csa5M601620.1	Ubiquitin-protein ligase
65	SNP	Csa5M602140.1	No hits found
66	SNP	Csa6M148340.1	No hits found
67	SNP/Indel	Csa6M497020.1	Unknown protein
68	SNP	Csa6M521050.1	Unknown protein
69	SNP	Csa7M023920.1	Predicted protein
70	SNP	Csa7M031580.1	RING-H2 finger protein ATL3J
71	SNP	Csa7M258340.1	Phosphatidylinositol 4-kinase
72	SNP/Indel	Csa7M322600.1	Proline-rich extensin-like family protein
73	SNP	Csa7M398120.1	Glycine-rich protein 5
74	Indel	Csa1M070580.1	Predicted protein
75	Indel	Csa2M034590.1	Kelch repeat-containing protein
76	Indel	Csa2M252060.1	No hits found
77	Indel	Csa3M026160.1	Hypothetical protein
78	Indel	Csa3M122570.1	No hits found
79	Indel	Csa3M568330.1	No hits found
80	Indel	Csa3M645890.1	Zinc finger protein
81	Indel	Csa3M702620.1	Proline-rich family protein
82	Indel	Csa3M748200.1	Late embryogenesis abundant protein
83	Indel	Csa4M268110.1	Emb2734 (embryo defective 2734)
84	Indel	Csa4M575360.1	No hits found
85	Indel	Csa5M321500.1	No hits found
86	Indel	Csa5M505760.1	DNA topoisomerase 1
87	Indel	Csa5M523120.1	Pentatricopeptide (PPR) repeat-containing protein
88	Indel	Csa5M576800.1	Mitochondrial import inner membrane translocase subunit tim44
89	Indel	Csa5M601520.1	Hypothetical protein
90	Indel	Csa6M092520.1	Unknown protein
91	Indel	Csa6M235580.1	No hits found
92	Indel	Csa6M409960.1	No hits found
93	Indel	Csa7M336510.1	ABA-insensitive 3
94	Indel	CsaUNM030560.1	No hits found

^a^Type of variant between D8 and SSL508-28; SNP/indel represents non-synonymous/frameshift indel

^b^Gene name

^c^Functional annotation of gene

**Table 5:** Predicted genes with NBS and LRR domains.

#	Genes	Predicted functions	E value	9930 V2.0 start	9930 V2.0 end
1	Csa2M435460.1	disease resistance protein (NBS-LRR class), putative	1.00E−72	22,802,549	22,805,481
2	Csa5M114640.1	leucine-rich repeat transmembrane protein kinase	3.00E−166	2,991,300	2,993,975
3	Csa5M495930.1	disease resistance protein (TIR-NBS-LRR class), putative	2.00E−07	17,398,520	17,398,969
4	Csa5M579010.1	TIR-NBS-LRR disease resistance protein	5.00E−21	20,457,095	20,457,578
5	Csa5M579560.1	disease resistance family protein / LRR family protein	3.00E−194	20,494,795	20,497,080

### Expression Analysis of Five Candidate Genes in Response to PM Inoculation

**Fig 7:**
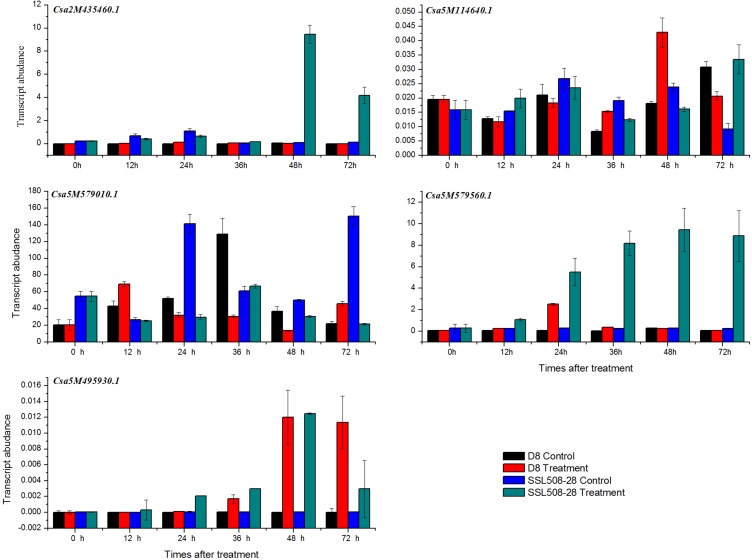
Expression level of 5 candidate genes in D8(susceptible, black and blue) and SSL508-28(resistant, red and dark cyan). Control means not inoculated with powdery mildew, treatment means inoculated with powdery mildew. The cucumber β-actin gene was used as an internal control, each value denotes the mean relative level of expression of three replicates.

qRT-PCR was performed to investigate the expression of five genes in SSL508-28 and D8, 0, 12, 24, 36, 48, and 72 h after inoculation with PM. The primers used are listed in S1 Table and expression levels of the five genes are presented in Fig 7. Two of these genes, *Csa2M435460*.*1* and *Csa5M579560*.*1*, showed low expression levels in D8 before and after inoculation, and in SSL508-28 before inoculation; but at 12 h after inoculation, both genes showed a marked upregulation. The expression level of *Csa5M579560*.*1* increased rapidly, and continued rising after inoculation at 0, 12, 24, 36, and 48 h, then decreased at 72 h, and *Csa2M435460*.*1* expression increased until 36 h after inoculation. Expression of the other three genes showed no regular trends between SSL508-28 and D8 (Fig 7).

### qRT-PCR Analysis of Candidate Genes in Different Cucumber Lines

**Fig 8:**
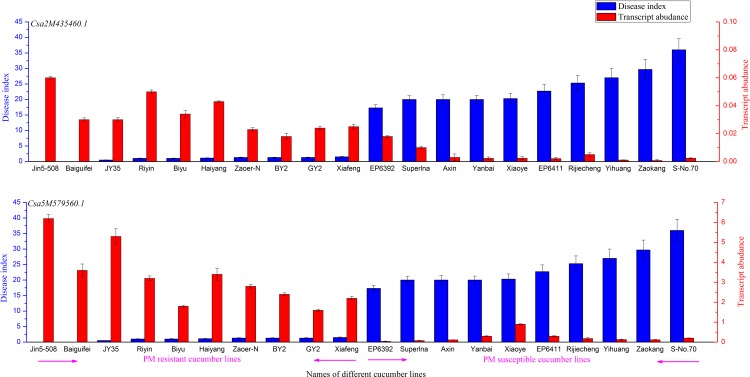
qRT-PCR analysis of candidate genes in different cucumber lines. The abscissa was the name of different cucumber lines, the left ordinate represents disease index of each cucumber lines, the right ordinate represents the expression level of candidate genes in each cucumber lines.

To investigate the expression levels of *Csa2M435460* and *Csa5M579560*.*1* in other cucumber lines, from 80 different cucumbers of our lab we selected 10 resistant and 10susceptible cucumber lines for qRT-PCR analysis after PM inoculation. Both genes were more highly expressed in the resistant lines than in the susceptible lines, with the donor parent JIN5-508 showing the highest expression (Fig 8). This provides further evidence for the putative roles of *Csa2M435460*.*1* and *Csa5M579560*.*1* in cucumber PM resistance.

### LRR Domain and Amino Acid Mutations of Two Candidate Genes Identified in D8 and SSL508-28

**Fig 9:**
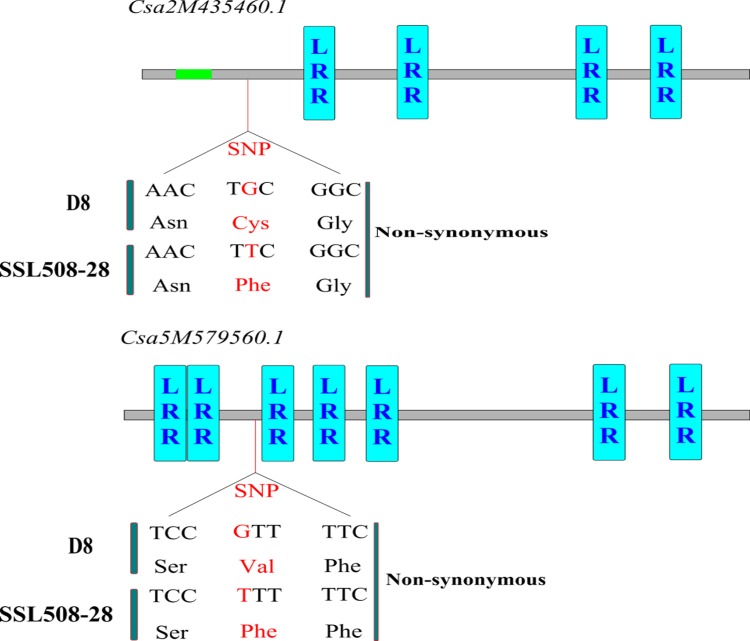
The mutation model of the Csa2M435460.1 and Csa5M579560.1. The red vertical lines stand for the SNP mutation site. The red amino acid sequences represent the amino acid site which has a non-anonymous SNP. Exons are shown by grey boxes, introns are shown by green boxes and the LRR domain was showed by blue boxes.

The protein sequences of *Csa2M435460*.*1* and *Csa5M579560*.*1* were extracted from the reference sequence (9930, Version 2.0) and analyzed in Pfam version 26.0. SMART was used to confirm the LRR domains. *Csa2M435460*.*1* and *Csa5M579560*.*1* encoded four and six LRR domains, respectively. To verify the presence of the non-synonymous SNPs in *Csa2M435460*.*1* and *Csa5M579560*.*1* in D8 and SSL508-28, the two genes were amplified in each line. The primers used are listed in S2 Table. Amplicon resequencing showed that the sequences of both genes in D8 were identical to those in SSL508-28 except at a single nucleotide: a G to T transversion caused a codon change of TGC to TTC in *Csa2M435460*.*1*, and a codon change of GTT to TTT in *Csa5M579560*.*1*, resulting in a missense, non-synonymous substitution of cysteine and valine for phenylalanine (Fig 9).

## Discussion

In this study, we used the SSL508-28 cultivar to identify PM resistance genes in cucumber. Different populations have previously been used in similar investigations [1, 3–7], including F2 and F2:3 populations or recombinant inbred lines, a significant limitation of these populations is the high amount of genetic background noise, which can mask the true effects of the QTL [36]. The SSL508-28 line we used in the present study possessed a segment from a PM-resistant donor, JIN5-508, which had a simpler genetic background than other populations, allowing more precise identification of the PM resistance genes [37]. According to the screening tests and plant height measurement over three seasons, SSL508-28 had a low disease index (Figs 1 and 2), indicating that SSL508-28 must be carried with PM resistance genes from the donor JIN5-508, but with the D8 genetic background. This suggested that the genetic variation between SSL508 and D8 was the main cause of the PM resistance phenotype [20]. Genome analysis based on resequencing of D8 and SSL508-28 was used for comprehensive identification of SNPs and indel variations to detect PMR-related genes. A total of 468,616 SNPs and 67,259 indels were detected in SSL508-28, and 537,352 SNPs and 91,698 indels were detected in D8 (Tables 2 and 3). Additionally, 15,682 SNPs and 6,262 indels were identified between SSL508-28 and D8. Annotating these SNPs and indels showed that there were only 120 non-synonymous SNPs and 30 frameshift indels, representing 94 genes, between SSL508-28 and D8 (Fig 6, Table 4), indicating that the SSL508-28 background was highly similar to D8 and that the phenotypic trait difference in PM infection is likely to be associated with these genes. Of the 94 genes predicted, four with NBS-LRR domains and one with an LRR domain were selected for qRT-PCR expression analysis (Table 5). *Csa2M435460*.*1* and *Csa5M579560*.*1* were revealed as the most likely candidate genes for resistance to PM because their expression increased in SSL508-28, but not D8, after inoculation with the PM pathogen (Fig 7). These genes were also more highly expressed in resistant versus susceptible cucumber lines (Fig 8). Both *Csa2M435460*.*1* and *Csa5M579560*.*1* contained the LRR domain (Fig 9), which is fundamental for the specific recognition of pathogen effectors in plants [38]. LRR proteins are involved in protein–ligand and protein–protein interactions that include the plant immune response [39], which is involved in disease resistance in many plant species including rice, tomato and *Arabidopsis thaliana* [40–42]. Indeed, more than 700 LRR domain-containing proteins have been identified in *Arabidopsis thaliana*, and 1400 in rice (http://www.ebi.ac.uk/interpro/).In cucumber, the LRR domain-containing gene *Rcy1* was found to be the primary specificity determinant for recognition of the yellow strain of the cucumber mosaic virus [43]. In wheat, *TmMla1*, which also contains an LRR domain, confers resistance to a strain of PM [18]. Similarly, in the present study, we show that two LRR protein genes confer PM resistance in cucumber, providing further evidence for the essential role of the LRR domain in host resistance against fungal pathogens. If PM resistance in cucumber is controlled by the LRR-type R-gene, this may indicate that LRR cluster members condition resistance to multiple diseases.

To validate the non-synonymous SNPs in *Csa2M435460*.*1* and *Csa5M579560*.*1* between SSL508-28 and D8, we cloned and sequenced the two genes in each line. In this way, we identified a non-synonymous SNP in each gene: a single G to T transversion caused a codon change of TGC to TTC in *Csa2M435460*.*1*, resulting in a non-synonymous cysteine to phenylalanine substitution. Furthermore, in *Csa5M579560*.*1*, a GTT to TTT codon change caused valine to change to phenylalanine (Fig 9).

The phenylalanine metabolic pathway is known to be important in plant disease resistance. Phenylalanine ammonia lyase activity increases in barley leaves and in cucumber seedlings after inoculation with PM [44, 45]. The pathway can also give rise to a wide range of secondary metabolites, such as flavonoids, catecholamines, phenolic and phenylpropanoic acids, phenols, lignins, and tannins [46]. The activity of phenylalanine ammonia lyase and its secondary metabolites should be investigated in D8 and SSL508-28 after infection with PM.

PM is a major disease that damages cucumber production around the world. It is important to discover the genes that confer resistance to this pathogen, and to understand the mechanisms underlying PM resistance. Here, using whole-genome sequencing, we have identified two PM resistance-related LRR-domain genes, located on chromosomes 2 and 5. The contribution of *Csa2M435460*.*1* and *Csa5M579560*.*1* to PM resistance in cucumber merits further investigation; indeed, functional validation studies of these two genes are ongoing.

## Conclusions

In the present study, the genomes of the cucumber cultivar SSL508-28 and its recipient parent, D8, were resequenced using the IlluminaHiSeq 2500 platform. Whole genome analysis revealed 15,682 SNP and 6,262 indel variations between SSL508-28 and D8. Of these, 120 non-synonymous SNPs and 30 frameshift indels in 94 genes were detected between the two lines. Among these 94 genes, five with an LRR or NBS-LRR domain were selected for qRT-PCR after PM pathogen inoculation in SSL508-28 and D8, which revealed *Csa2M435460*.*1* and *Csa5M579560*.*1* as the most likely candidate genes. Subsequent qRT-PCR analysis of *Csa2M435460*.*1* and *Csa5M579560*.*1* in multiple resistant and susceptible cucumber lines confirmed that *Csa2M435460*.*1* and *Csa5M579560*.*1* are involved in resistance to PM. Mutations in these genes may be a major cause of the high PM resistance in the SSL508-28 line. The present findings are a promising step towards improving PM-resistant cucumber breeding.

## Supporting Information

S1 Table Primers for qRT-PCR analysis of candidate genes response to PM inoculation. (DOCX)

S2 Table Primers for *Csa2M435460*.*1* and *Csa5M579560*.*1* cloning. (DOCX)
