# Supplementary material for: Whole-Genome Resequencing of a Cucumber Chromosome Segment Substitution Line and Its Recurrent Parent to Identify Candidate Genes Governing Powdery Mildew Resistance
Source: PLoS One. 2016 Oct 20;11(10):e0164469. doi: 10.1371/journal.pone.0164469 (PMC5072683; doi:10.1371/journal.pone.0164469)
Supplement: S1 Table — (DOCX) [file pone.0164469.s001.docx]

**S1 Table: Primers for qRT-PCR analysis of candidate gene response to PM inoculation**

| Genes | Forward primer (5’–3’) | Reverse primer(5’–3’) | Product (bp) |
| --- | --- | --- | --- |
| Csa2M435460.1 | AGATGGGAGGAAATGTTG | GCAGATGACGGTAGACGA | 189 |
| Csa5M114640.1 | TCTTTGGGAGATTTGGTT | AATGGAGGGTGAAGTGAT | 197 |
| Csa5M495930.1 | TCAAACAAAGCCTCCCTC | TCCCACGTTATGCAAACT | 134 |
| Csa5M579010.1 | TGTATCTAAGAGGGTGCG | CCCGAAAGACAGAACGAA | 162 |
| Csa5M579560.1 | GCTGTAACGGAGATTGGG | TTGAACCGCTGATATGCT | 187 |
| Actin | TCGTGCTGGATTCTGGTG | GGCAGTGGTGGTGAACAT | 136 |
