# Supplementary material for: Whole-Genome Resequencing of a Cucumber Chromosome Segment Substitution Line and Its Recurrent Parent to Identify Candidate Genes Governing Powdery Mildew Resistance
Source: PLoS One. 2016 Oct 20;11(10):e0164469. doi: 10.1371/journal.pone.0164469 (PMC5072683; doi:10.1371/journal.pone.0164469)
Supplement: S2 Table — (DOCX) [file pone.0164469.s002.docx]

**S2 Table: Primers for *Csa2M435460.1* and *Csa5M579560.1* cloning**

| Genes | Forward primer (5’–3’) | Reverse primer (5’–3’) |
| --- | --- | --- |
| Csa2M435460.1 | ATGAAGAAGGTGTGTGATTTC | CACTTCTGATAACCGTTGCGC |
| Csa5M579560.1 | ATGCAGTTTCTCAAATGG | GTCTTCTTTTTCGATTCT |
